# The Antitumor Activity of a Lead Thioxanthone is Associated with Alterations in Cholesterol Localization

**DOI:** 10.3390/molecules23123301

**Published:** 2018-12-12

**Authors:** Raquel T. Lima, Diana Sousa, Ana Sara Gomes, Nuno Mendes, Rune Matthiesen, Madalena Pedro, Franklim Marques, Madalena M. Pinto, Emília Sousa, M. Helena Vasconcelos

**Affiliations:** 1 i3S-Instituto de Investigação e Inovação em Saúde, Universidade do Porto, Rua Alfredo Allen 208, 4200-135 Porto, Portugal; dsousa@ipatimup.pt (D.S.); nmendes@ipatimup.pt (N.M.); 2 Cancer Drug Resistance Group—IPATIMUP—Institute of Molecular Pathology and Immunology of the University of Porto; Rua Júlio Amaral de Carvalho, 45, 4200-135 Porto, Portugal; 3 Department of Pathology, Faculty of Medicine of the University of Porto, Alameda Prof. Hernâni Monteiro, 4200-319 Porto, Portugal; 4 Laboratory of Microbiology, Department of Biological Sciences, FFUP-Faculty of Pharmacy, University of Porto, Rua de Jorge Viterbo Ferreira 228, 4050-313 Porto, Portugal; anasarag4@gmail.com; 5 UCIBIO, REQUIMTE, Laboratory of Biochemistry, Department of Biological Sciences, Faculty of Pharmacy, University of Porto, Rua de Jorge Viterbo Ferreira, 228, 4050-313 Porto, Portugal; franklim@ff.up.pt; 6 HEMS-Histology and Electron Microscopy-i3S, Rua Júlio Amaral de Carvalho, 45, 4200-135 Porto, Portugal; 7 Computational and Experimental Biology Group, The Chronic Diseases Research Center (CEDOC), Nova Medical School, Faculdade de Ciencias Medicas Universidade Nova De Lisboa, Rua Câmara Pestana 61150-082 Lisboa, Portugal; rune.matthiesen@nms.unl.pt; 8 CESPU, Instituto de Investigação e Formação Avançada em Ciências e Tecnologias da Saúde, IUCS-Instituto Universitário de Ciências da Saúde, Rua Central de Gandra 1317, 4585-116 Gandra, Portugal; madalena.oliveira.pedro@gmail.com; 9 Laboratory of Organic and Pharmaceutical Chemistry, Department of Chemical Sciences, FFUP-Faculty of Pharmacy, University of Porto, Rua de Jorge Viterbo Ferreira 228, 4050-313 Porto, Portugal; madalena@ff.up.pt (M.M.P.); esousa@ff.up.pt (E.S.); 10 CIIMAR/CIMAR—Centro Interdisciplinar de Investigação Marinha e Ambiental, Universidade do Porto, Terminal de Cruzeiros do Porto de Leixões, Avenida General Norton de Matos, S/N, 4450-208 Matosinhos, Portugal

**Keywords:** thioxanthones, cholesterol localization, tumor xenografts, non-small cell lung cancer, antitumor activity, rags

## Abstract

The search for novel anticancer small molecules and strategies remains a challenge. Our previous studies have identified TXA1 (1-{[2-(diethylamino)ethyl]amino}-4-propoxy-9H- thioxanthen-9-one) as a hit compound, with in vitro antitumor potential by modulating autophagy and apoptosis in human tumor cell lines. In the present study, the mechanism of action and antitumor potential of the soluble salt of this molecule (TXA1.HCl) was further investigated using in vitro and mouse xenograft tumor models of NSCLC. Our results showed that TXA1.HCl affected steroid biosynthesis, increased RagD expression, and caused abnormal cellular cholesterol localization. In addition, TXA1.HCl treatment presented no toxicity to nude mice and significantly reduced the growth of human NSCLC cells xenografts in mice. Overall, this work provides new insights into the mechanism of action of TXA1, which may be relevant for the development of anticancer therapeutic strategies, which target cholesterol transport.

## 1. Introduction

Cholesterol is an essential component at the basis of a wide array of cellular functions [1]. Mainly found at the plasma membrane, cholesterol plays a structural role in membrane fluidity and membrane’s protein activity [2]. Importantly, cholesterol is also fundamental for the biosynthesis of critical biochemical molecules, including steroid hormones, vitamin D, and bile acids [3]. Cholesterol cellular levels are highly controlled by a tightly regulated network of cell signaling and lipid transfer systems involving not only mechanisms for de novo synthesis and esterification but also synthesis of LDLR (low-density lipoprotein receptor) to internalize extracellular cholesterol [1,3,4].

Alterations in cholesterol homeostasis impact many key signaling pathways, namely cell growth and survival, thus being associated with several different pathologies, namely cancer [5]. Indeed, changes in cholesterol normal levels may be involved in carcinogenesis and tumor development in several cancers, acting in some cases as promoters, while in others as suppressors [5,6]. In addition to alterations in its cellular levels, perturbations in cholesterol localization or trafficking have been shown to affect cellular viability [7]. In fact, targeting cholesterol transport has been described as a potential therapeutic strategy, although it has not been clinically proven [7,8,9]. Leelamine, a tricyclic diterpene molecule extracted from the bark of pine trees, induced cell death in melanoma cells through the shutdown of cholesterol egress from lysosomal/late endosomal cell compartments [9,10]. Interestingly, studies on repurposing antipsycothic agents as novel anticancer agents have been published [11]; among those studies, drugs such as imipramine, olanzapine and pimozide are described as inducers of accumulation of cholesterol (although their effect on cholesterol biosynthesis is still a matter of study) [12,13,14]. Also, the antifungal agent itraconazole, has anticancer properties [15,16]; among several mechanisms of action already described, itraconazole decreased endothelial and glioblastoma cells viability (inducing autophagy) through disruption of cholesterol trafficking and reduction of its plasma membrane levels [15,17]. Furthermore, JNJ-26854165 [serdemetan, a human double minute protein (HDM)-2 antagonist] induced cell death and inhibited cholesterol transport in mantle cell lymphoma (MCL) and multiple myeloma (MM) [8].

We have previously shown that a synthetic hit thioxanthonic molecule (1-{[2-(diethylamino)ethyl]amino}-4-propoxy-9H-thioxanthen-9-one, TXA1) and its soluble salt (TXA1.HCl), reduced the in vitro growth of a panel of human tumor cell lines, without affecting the growth of non-tumor cells [18,19,20]. Such antitumor effect was shown to involve autophagy modulation in melanoma and breast cancer cells [20]. Nevertheless, the underlying mechanism of action of TXA1.HCl was not fully elucidated, nor was its antitumor potential in vivo verified. Therefore, in the current study, the effect of TXA1.HCl was investigated in cells from a non-small cell lung cancer (NSCLC) cell line, both in vitro and in mice xenografts. Our data showed that this thioxanthone induced an abnormal cellular cholesterol localization and reduced the growth of human NSCLC cells in vitro and of NSCLC xenografts in mice, without presenting toxicity to the animals.

This study gives new insights into the mechanism of action of a thioxanthone compound, here identified to be through the targeting of cholesterol transport, and demonstrated that this compound has in vivo efficiency and could be considered a lead compound.

## 2. Results and Discussion

### 2.1. TXA1.HCl Affects Steroid Biosynthesis and Cholesterol Localization

**Figure 1:**
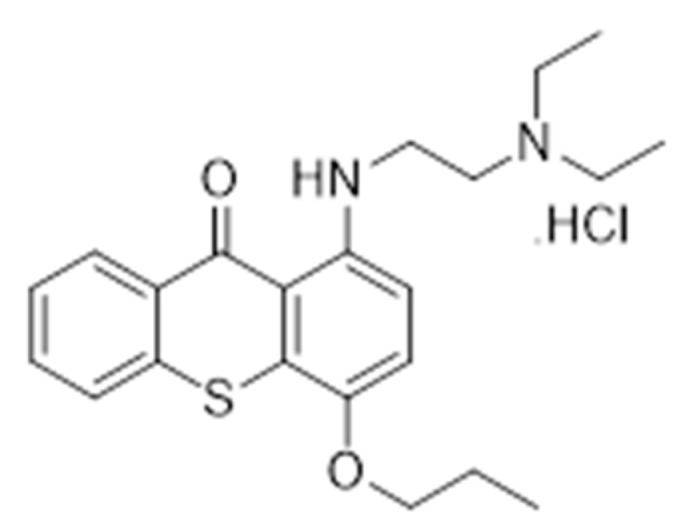
TXA1.HCl.

TXA1.HCl (Figure 1) has been described in our previous studies as a hit thioxanthonic compound, which decreased human tumor cells viability through a mechanism involving autophagy and apoptosis, without affecting the growth of non-tumor human cells [18,19,20].

**Figure 2:**
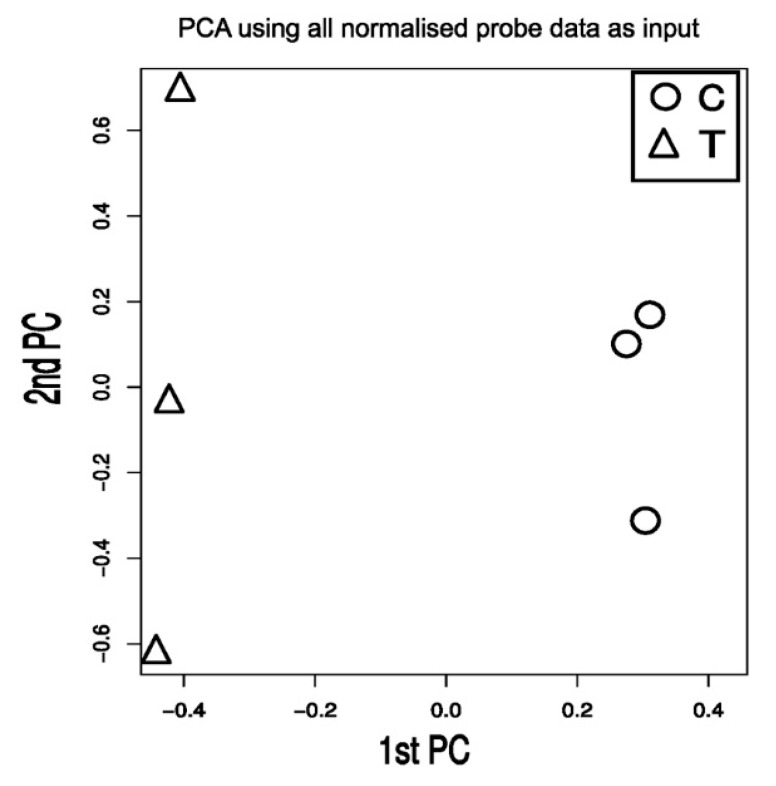
Basic overview of the microarray expression data. Principal component analysis using all measured genes expressed on the microarray as input.

To gain insight into the mechanism of action of TXA1.HCl, total RNA was extracted from NCI-H460 cells treated for 24 h with this compound (at 6.9 µM, previously determined GI_50_ concentration [18]) or from control cells (treated with H_2_O, vehicle). A cDNA microarray analysis of three independent biological replicates identified a total of 161 differentially expressed transcripts (>1.5-fold and with statistically significance, *p* < 0.05) between NCI-H460 cells treated with TXA1.HCl and controls. Among these, 133 transcripts were up-regulated and 28 were down-regulated in TXA1.HCl treated cells (Figure 2 and Supplementary Table 1). Data from the microarray data has been deposited in Gene Expression Omnibus (GEO) and are accessible through accession number GSE110825.

**Figure 3:**
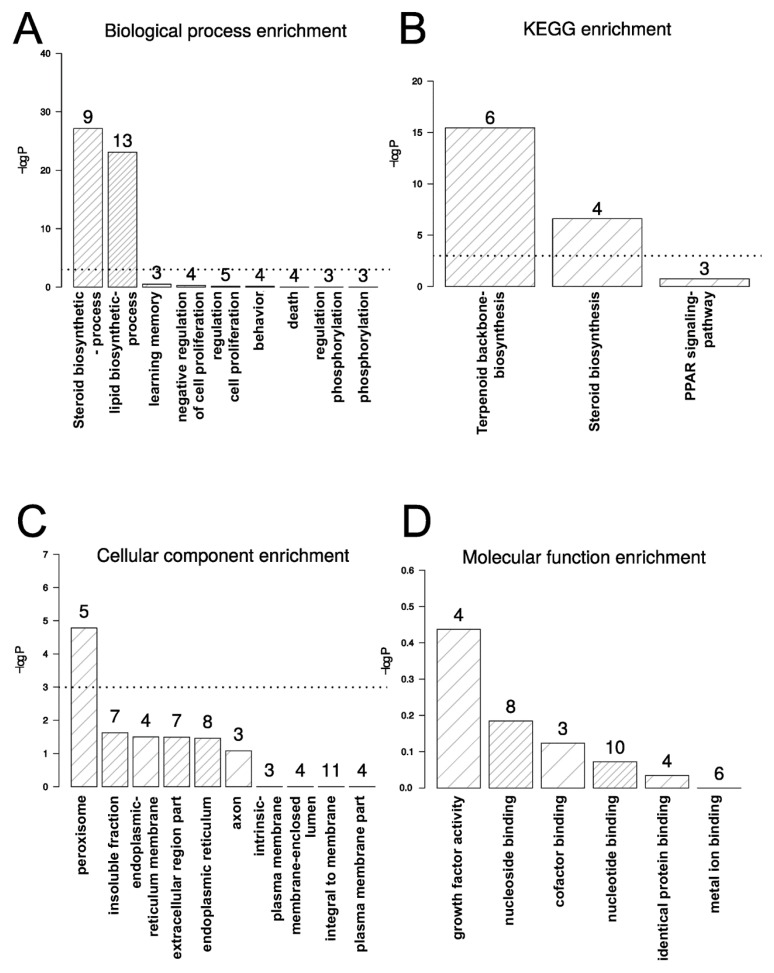
Histograms depicting the functional enrichment for: (**A**) biological processes, (**B**) KEGG, (**C**) cellular component and (**D**) molecular function. The numbers above each bar represent the number of expressed genes identified as being significantly regulated for each of the displayed functional categories. The dotted line indicates −log (0.05).

Analysis of the 133 overexpressed genes (>1.5-fold and adjusted p value less than 0.05) with the Database for Annotation, Visualization and Integrated Discovery (DAVID) network analysis tool, allowed separating the transcripts according to biological processes and pathways, cellular components and molecular functions (Figure 3). Interestingly, the biological processes and KEGG pathways [21,22,23] mainly affected by TXA1.HCl treatment were restricted only to two (overlapping) pathways: the terpenoid-backbone biosynthesis and the steroid biosynthesis pathways (Figure 3a,b).

**Figure 4:**
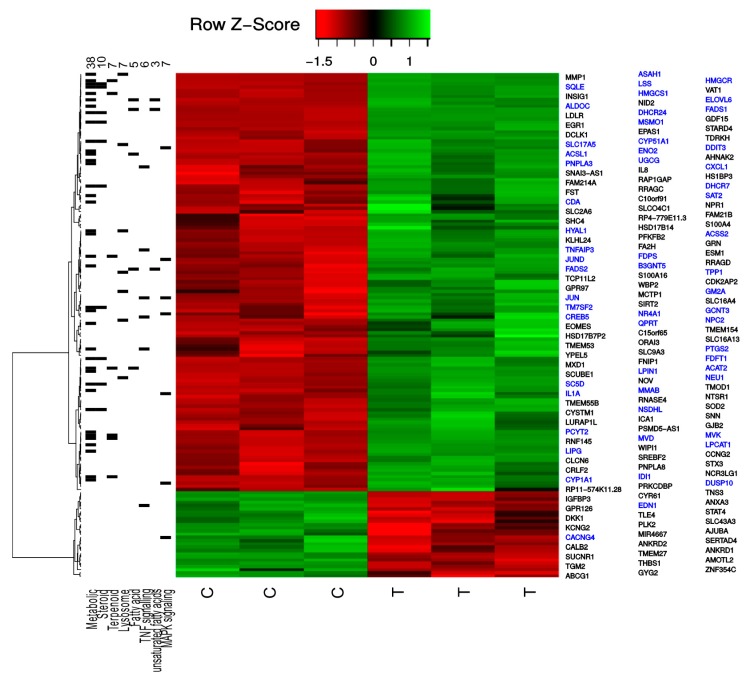
Heatmap depicting the normalized expression values for the significant regulated proteins with more than 1.5-fold, including the most enriched KEGG pathways. The left side indicates the expressed genes for each of the most enriched KEGG pathways. The left top numbers indicate the number of significantly altered mRNAs that was identified from each of the KEGG pathways. The genes highlighted in blue belong to at least one of the indicated KEGG pathways.

This result on the terpenoid-backbone biosynthesis was also confirmed by KEGG enrichment analysis using the CoFRA R package (Figure 4) [24].

Noteworthy, results from the cDNA microarray analysis also showed evident alterations in the expression of genes involved in the cellular trafficking of cholesterol, in NCI-H460 cells after TXA1.HCl treatment (Figure 4). Indeed, genes such as LDLR (low-density lipoproteins receptor, a cell surface receptor involved in LDL-cholesterol endocytosis) [25], NPC2 (NPC intracellular cholesterol transporter 2) and SREBF2 (sterol regulatory element-binding protein 2), were found upregulated. On the other hand, ABCG1 (an ABC transporter involved in cholesterol efflux) was downregulated after TXA1.HCl treatment (Figure 4 and Supplementary Table 1). These data, together with the increase in the cholesterol biosynthesis pathway referred above, indicates that the cellular availability of cholesterol was compromised when cells were treated with TXA1.HCl. 

**Figure 5:**
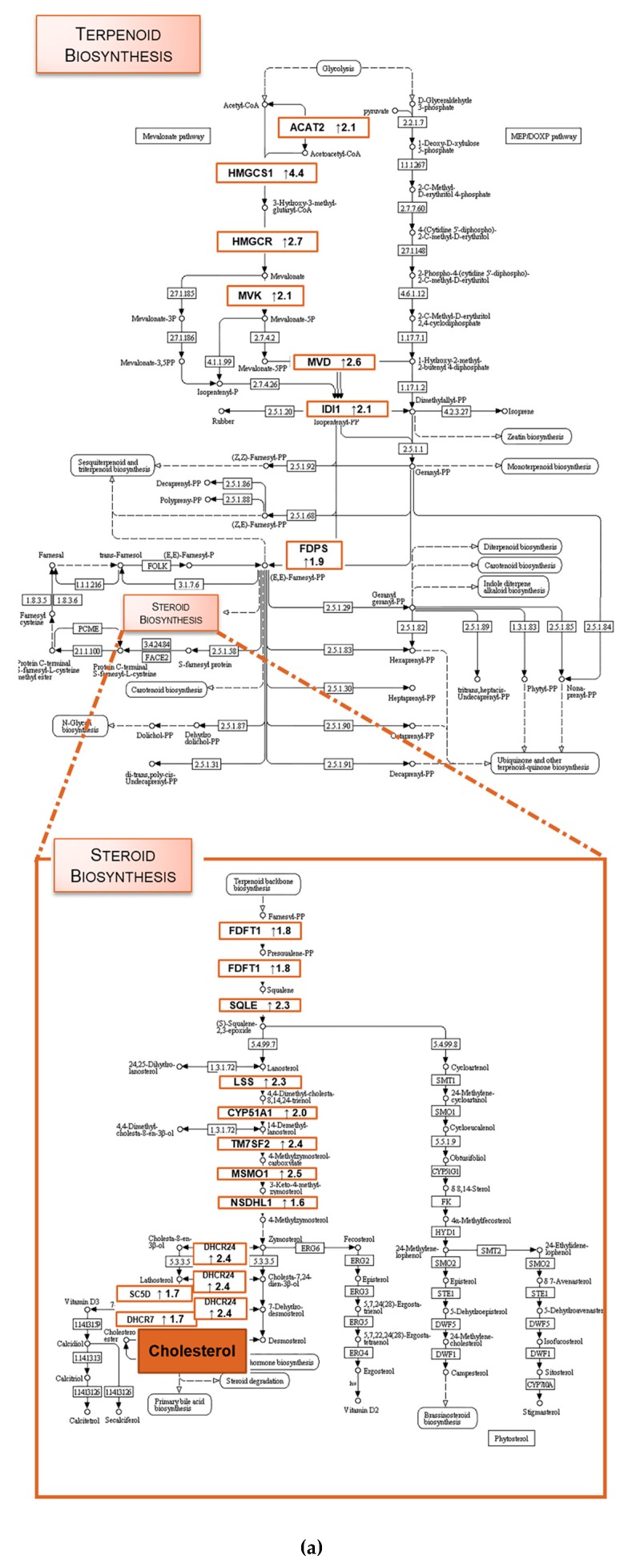

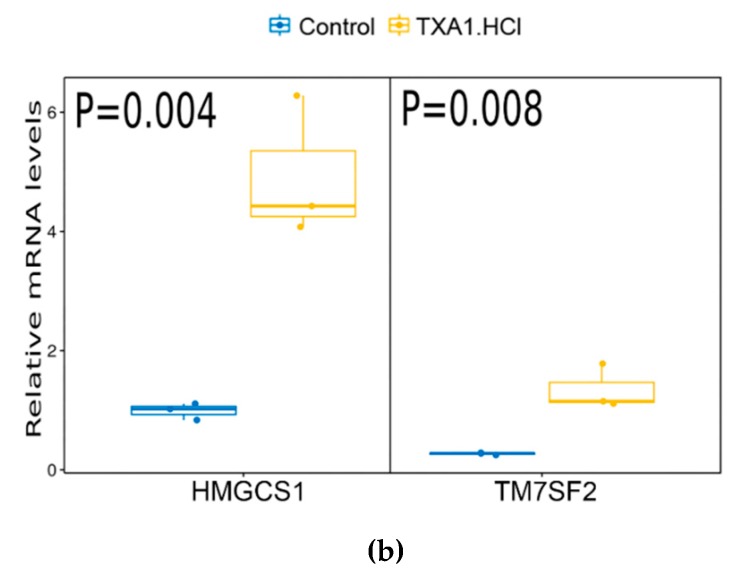
(**a**) KEGG pathways with significantly differential gene expression from this study were highlighted, including the direction of regulation (up or downregulation). Each colored box has the gene name abbreviated and a number representing the ≥1.5-fold relative to the control. KEGG pathway background images were adapted from www.genome.jp/kegg/ [21,22,23]. (**b**) HMGCS1 and TM7SF2 mRNA levels, analyzed by quantitative real-time PCR. Values are expressed after normalization for an endogenous control. Data plotted with ggplot and p value were calculated using *t*. test in R statistical programming language comparing TXA1.HCl vs control. Gene names: ACAT2, acetyl-coenzyme A acetyltransferase 2; HMGCS1, 3-hydroxy-3-methylglutaryl-coenzyme A synthase 1; HMGCR, 3-hydroxy-3-methylglutaryl-coenzyme A reductase; MVK, mevalonate kinase; MVD, mevalonate (diphospho) decarboxylase; IDI1, isopentenyl-diphosphate δ-isomerase 1; FDPS, farnesyl diphosphate synthetase; FDFT1, squalene synthase; SQLE, squalene epoxidase; LSS, lanosterol synthase; CYP51A1, cytochrome P450, family 51, subfamily A, polypeptide 1 (lanosterol 14 α-demethylase); TM7SF2, transmembrane 7 superfamily member 2 (Delta(14)-sterol reductase); MSMO1, methylsterol monooxygenase; NSDHL, NAD(P)-dependent steroid dehydrogenase; DHCR24, 24-dehydrocholesterol reductase; SC5DL, sterol-C5-desaturase-like; DHCR7, 7-dehydrocholesterol reductase.

The KEGG pathway analysis identified 17 non-redundant transcripts involved in terpenoid and steroid biosynthesis pathways (Figure 5 and Supplementary Table 1). All 17 transcripts were up-regulated in TXA1.HCl treated cells (1.6 to 4.4-fold), being 3-hydroxy-3-methylglutaryl-CoA synthase 1 (HMGCS1), the most up-regulated transcript (Figure 5 and Supplementary Table S1). The induction of enzymes associated with cholesterol biosynthesis, namely HMGCS1 and TMS7F2, was further validated by real-time quantitative PCR (Figure 5b). Together, these results suggest that TXA1.HCl caused an up-regulation of the biosynthesis of cholesterol.

**Figure 6:**
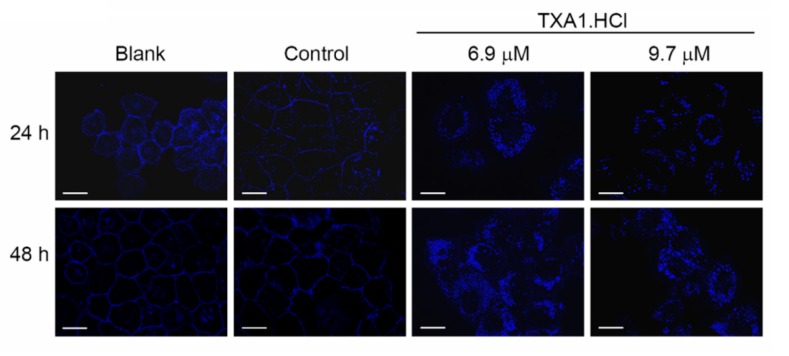
TXA1.HCl treatment affects cholesterol localization in vitro of NCI-H460 cells. NCI-H460 cells were treated with TXA1.HCl (6.9 µM and 9.7 µM) for 24 and 48 h, or with respective controls. Cholesterol localization was evaluated by filipin staining. Images are representative of three independent experiments. Bar = 20 µM.

Therefore, in order to confirm if the cellular availability of cholesterol was compromised, filipin (a fluorescent staining of unesterified cholesterol) was used to visualize cholesterol in NCI-H460 cells following treatment with TXA1.HCl for 24 or 48 h (Figure 6). While in untreated and control cells, cholesterol was predominantly distributed at the plasma membrane, TXA1.HCl treated cells showed a clear reduction of membrane-associated cholesterol with a concomitant increase in the punctate pattern for the filipin staining in the cytoplasm (Figure 6). This was observed not only after treatment with 6.9 µM TXA1 (GI_50_) but also with a higher TXA1.HCl concentration (9.7 µM, corresponding to approximately the GI_75_, concentration which decreases cell viability to 25%, Supplementary Figure S1). These results indicate that TXA1.HCl induces alterations in cholesterol cellular distribution, potentiating its cytoplasmic accumulation. 

### 2.2. TXA1.HCl Induces Autophagy and Decreases Proliferation of NSCLC Cells

**Figure 7:**
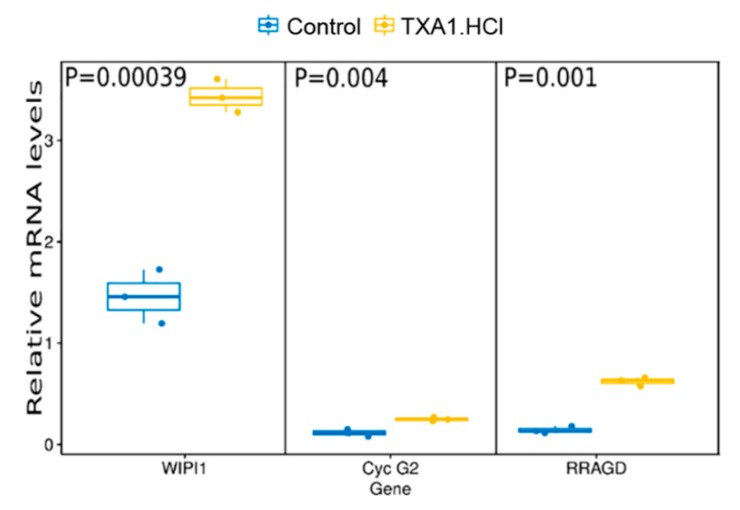
TXA1.HCl treatment affects WIPI1, Cyclin G2 and RRAGD mRNA expression levels, confirmed by quantitative real-time PCR. Values are expressed after normalization for an endogenous control (Hprt1). Data plotted with ggplot and *p* values were calculated using *t*. test in R statistical programming language, comparing TXA1.Hcl treatment vs control.

In our previous studies, we demonstrated that TXA1 decreased breast cancer and melanoma cells viability through a mechanism involving autophagy modulation (at the GI_50_ concentration) and increased apoptosis (at concentrations higher than GI_50_) [18,20]. Interestingly, it has been described that changes in the cellular lipid environment may induce autophagy. This was shown not only in cases of cellular cholesterol depletion but also when cholesterol trafficking is abnormal [26,27]. Furthermore, cholesterol trafficking seems to be important for mTOR activation [28], which negatively regulates autophagy [29,30]. Accordingly, in the present work, further analysis of the list of transcripts that were differentially expressed following TXA1.HCl treatment, indicated that some of the significantly overexpressed genes were associated with autophagy and/or mTOR signaling (Figure 4 and Figure 7, and Supplementary Table S1). NPC2, involved in the egress of cholesterol from the lysosome [31], was shown to induce autophagy and to inhibit cell growth in a murine breast cancer cell model [32] while its deficiency impaired autophagy-lysosomal activity in adipocytes [33]. WIPI1, belonging to the WD-repeat protein interacting with the phosphoinositides (WIPI) family, is necessary for autophagosome formation and its increased mRNA levels has been introduced as a possible biomarker of autophagosome formation [34,35]. Expression of the unconventional cyclin G2 encoded by CCNG2 has been previously shown to increase following treatment with an mTOR inhibitor, rapamycin [36,37], and to induce autophagy in chronic myeloid leukemia cells [38]. Likewise, RRAGD (and RRAGC) are members of the Rag family of GTPases described to recruit mTORC1 to the lysosomal surface, induced by the presence of amino-acids, crucial for its subsequent activation by Rheb GTPase [39,40,41,42].

**Figure 8:**
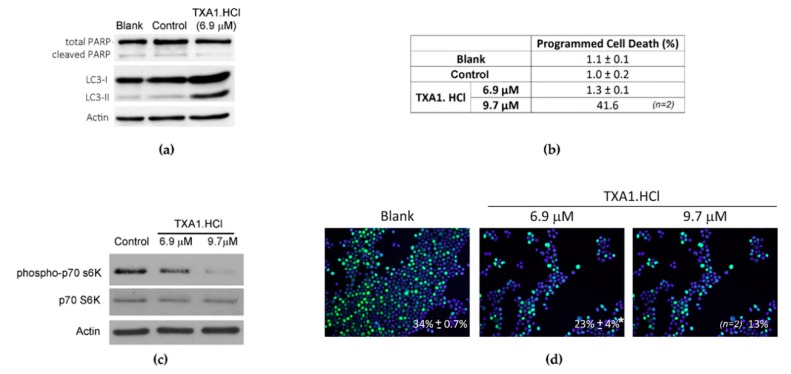
TXA1.HCl treatment induced autophagy. NCI-H460 cells were treated with 6.9 µM or with 9.7 µM TXA1.HCl for 48 h or with the solvent control (H_2_O). (**a**) PARP-1 and LC3 levels were analyzed by Western blot. Actin was used as loading control. (**b**) Programmed cell death was assessed with the TUNEL assay. (**c**) Levels of p70 s6K protein and of the phosphorylated form were analyzed by Western blot. Actin was used as a loading control. (**d**) Cellular proliferation was evaluated with the BrdU incorporation assay followed by fluorescence microscopy. In blue, Dapi stained nuclei. In green, BrdU incorporating nuclei. Values in the images correspond to the % of proliferating cells, expressed as mean ± SEM (* *p* < 0.05 vs. control). Results (and representative images) are from at least 3 independent experiments, except when otherwise stated (which were from 2 experiments only).

Furthermore, modulation of autophagy by TXA1.HCl was also confirmed in the present study by the increased LC3-II levels following cellular treatment with 6.9 µM TXA1.HCl (Figure 8a). This concentration had no effect on cellular apoptosis, since there was no increase in PARP cleavage and no positive results in the TUNEL assay (Figure 8a,b). Moreover, since inhibition of the mTOR pathway is one of the main signals for autophagy induction [29,30], the phosphorylation status of its downstream p70 S6K protein was analyzed, as a readout of the pathway activity (Figure 8c). Results showed that TXA1.HCl treatment induced a clear p70 S6K protein dephosphorylation, suggesting that the effect of TXA1.HCl involved, at least partially, the mTOR pathway. In addition, a statistically significant decrease in the NCI-H460 cellular proliferation rate, analyzed with the BrdU incorporation assay, was observed following TXA1.HCl treatment (Figure 8d). This effect may be associated with the inhibition of the mTOR signaling pathway, by this compound [43]. Indeed, mTOR is known to regulate cell growth and proliferation in response to a wide range of signals [44,45].

Effects on proper cholesterol distribution to the plasma membrane (and likely to other intracellular membranes) has been described for other compounds, which affect the viability of cancer cells [8,17,46], some involving autophagy [25,46]. Nevertheless, the effect of these compounds on steroid synthesis may differ. Indeed, our study showed that TXA1.HCl induced autophagy and abnormal cholesterol trafficking while increasing cholesterogenic enzymes. This had been previously observed in cancer cells treated with TAIII (timosaponin-AIII, isolated from the medicinal herb Anemarrhena asphodeloides) [46], in which high cholesterol content could be supporting autophagy through its incorporation in the autophagic vacuole membranes [46,47]. On the other hand, itraconazole, which is a widely used antifungal agent, induced autophagy in cancer cells by affecting normal cholesterol distribution although causing a decrease in cholesterol biosynthesis [17]. Nevertheless, several mechanisms of action have been described for itraconazole [15,16], namely that it represses lanosterol 14-alpha-demethylase which is critical for cholesterol biosynthesis, thus justifying the reduction in cellular levels of cholesterol.

### 2.3. TXA1.HCl Reduces the Growth of NCI-H460 Xenografts in Nude Mice

**Figure 9:**
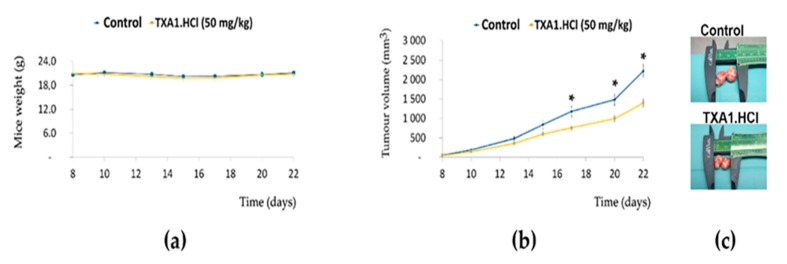

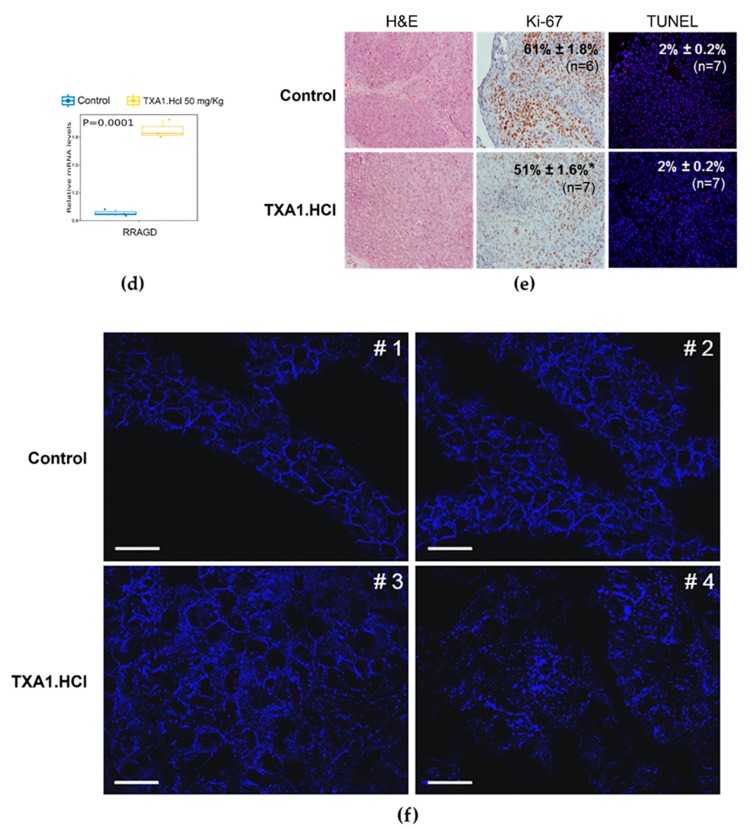
TXA1.HCl decreases tumor growth and proliferation and affects cholesterol localization and RAGD expression in NCI-H460 tumor xenografts in nude mice. Nude mice bearing tumor xenografts (40–80 mm^3^) were *s.c.* injected three times per week with TXA1.HCl (50 mg/kg, *n* = 7) or vehicle control (saline, *n* = 7). (**a**) Body weight changes in mice during treatment. Results are expressed as the mean ± SEM, *n* = 7. * *p* < 0.05 vs. control. (**b**) Tumor growth curves. Tumor dimensions were measured using calipers and volumes inferred on the indicated days as previously mentioned. (**c**) Representative tumors resected at the end of the experiment (at day 22 after cells inoculation). Caliper is present to compare the tumor sizes. Since some tumors were bilobulated, each lobule was measured alone and the total volume was inferred. (**d**) Tumors histological analysis. Representative images of tumor xenografts sections: H&E stain, immunohistochemistry for Ki-67 and TUNEL fluorescence microscopy. Original magnification is 40x. (**e**) RagD expression levels, analyzed by quantitative real-time PCR. Values are expressed after normalization for an endogenous control (Hprt1). Data plotted with ggplot and p values were calculated using *t*. test in R statistical programming language, comparing TXA1.Hcl treatment *vs* control. Results are expressed as mean ± SEM, for *n* = 3. * *p* < 0.05 vs. control. (**f**) Cholesterol localization was evaluated by filipin staining. Images are representative of tumor xenografts from the different animals (control mice #1 and #2; treated mice #3 and #4). Bar = 20 µM.

The toxicity and antitumor potential of TXA1.HCl were further assessed in vivo, using nude mice. First, a preliminary study was performed to assess toxicity of TXA1.HCl by injecting subcutaneously (*s.c.*, three times per week) increasing doses of TXA1.HCl for two weeks. Since no significant discomfort signs were observed during treatment (data not shown), the maximum dose (50 mg/kg) was chosen to proceed the study in NCI-H460 cells xenografted mice. For this, NCI-H460 cells were injected *s.c.* in the flanks of nude mice and tumor xenografts were allowed to grow. Once tumors reached 40–80 mm^3^, mice were injected with vehicle control (saline) or with TXA1.HCl (50 mg/kg). As expected, throughout the duration of the treatment, TXA1.HCl treated xenografted mice and control mice showed no significant discomfort signs (including significant alteration in body weight) (Figure 9a).

Furthermore, the evaluation of biochemical and toxicological parameters in serum samples from TXA1.HCl treated and control mice showed no signs of toxicity (no statistically significant differences were found, Table 1).

Importantly, tumor growth was significantly delayed by TXA1.HCl throughout the duration of the experiment (22 days), particularly from day 17 after inoculation of the tumor cells into the mice (Figure 9b). At the end of the experiment (day 22), the mean tumor volume measured in the mice flanks was 1399 ± 101 mm^3^ in the treated group and 2218 ± 165 mm^3^ in the vehicle control group (Figure 9b,c). In addition, analysis of the total tumor weight of TXA1.HCl-treated tumors after resection showed a decrease when compared with the controls (Supplementary Figure S2). These results showed that treatment of mice with 50 mg/kg of TXA1.HCl resulted in reduction of tumor growth in vivo, without causing toxicity.

### 2.4. TXA1.HCl Treatment Reduces Proliferation, Increases RagD Expression and Affects Cholesterol Localization in NSCLC Xenografted Nude Mice

To further confirm the effect of TXA1.HCl in vivo, tumor xenografts were extracted and further analyzed. TXA1.HCl treatment caused no obvious signs of oncocytic necrosis or fibrosis, as could be observed following H&E staining of the removed xenografted tumors (Figure 9d). NCI-H460 xenografts from TXA1.HCl treated mice had less Ki67 staining (Figure 9d) when compared to the control group, demonstrating that TXA1.HCl reduced proliferation in vivo. This evidence was in accordance with the results previously obtained in vitro, in which decreased proliferation levels were observed (by the BrdU incorporation assay, Figure 8d) following TXA1.HCl treatment. This decreased proliferation could be associated with inhibition of mTOR by TXA1.HCl, as previously shown (Figure 8c). Also, microarrays results had shown an increase in Cyclin G2 levels after treatment with TXA1.HCl. This unconventional cyclin, which is also negatively regulated by mTOR, has been previously described to inhibit cellular proliferation [37,38]. Identical (low) levels of apoptosis were observed (using TUNEL assay) in the TXA1.HCl treated xenografts and in the control tumors (Figure 9d).

In addition, analysis of RRAGD (or RAGD) mRNA levels in the xenografted tissues showed a significant upregulation of this mRNA in TXA1.HCl treated mice, when compared to the controls, in agreement with the data previously observed in vitro. However, no statistically significant alterations were confirmed in vivo for the other genes that had previously been identified in vitro (data not shown). Although a very recent study showed that induction of RagD expression controlled the mTORC1 lysosomal recruitment and activity [48], the upregulation of RAGD, particularly by TXA1.HCl treatment, still needs to be fully explored. Interestingly, and as previously referred, these proteins belong to the family of Ras related GTPases (Rags), mainly described to activate mTOR through its recruitment to the lysosomal surface in the presence of aminoacids (rather than through direct mTOR kinase activation) [44,49,50]. The Rag protein family is composed of four members (A to D). Only RagD (and -C) were found differentially expressed in the present work. Interestingly, RagC/D share high aminoacid homology, being functionally redundant, and form heterodimers with Rag A/B (which also have high aminoacid homology and are functionally redundant), in an active conformation [51,52]. In addition, in the most active conformation, Rag A/B bind to GTP while Rag C/D bind to GDP [51,52,53]. This heterodimer active conformation physically interacts with and recruits mTORC1 (by binding Raptor) to the surface of lysosomes, where it is activated by a small GTPase named Rheb [41,42]. Although our results show an increase in Rags C/D following TXA1.HCl treatment, it remains to be clarified if and how TXA1.HCl treatment may interfere with the Rags C/D GTP/GDP status and with its balance with Rags A/B. Moreover, even though Rags activity has been mainly associated with aminoacids stimuli [40,54], Rags GTPases were recently found to transmit information from other nutrient signals [53,55,56], namely cholesterol [57].

Finally, since the in vitro studies had shown that TXA1.HCl treatment affected cholesterol localization, filipin staining was carried out in tumor xenograft sections to evaluate if the same was detected in vivo. Alterations in the membrane pattern of cholesterol localization were clearly observed (Figure 9f). When analyzing xenograft tumor samples from different mice following treatment, a clear alteration in the localization of cholesterol was observed in the samples from TXA1.HCl treated mice. Indeed, while in control treated tumors, the cholesterol staining was highly homogenous and membrane-associated, in TXA1.HCl treated tumors, the pattern of cholesterol was heterogeneous, with a decrease in the membrane pattern and an increase in the cytoplasm. This was in agreement with the in vitro results obtained above, further confirming that TXA1.HCl induces alterations in cholesterol localization.

## 3. Materials and Methods 

### 3.1. Synthesis of TXA1 Hydrochloride (TXA1.HCl).

1-{[2-(Diethylamino)ethyl]amino}-4-propoxy-9H-thioxanthen-9-one (TXA1) was synthesized, as previously described [18]. An etheric solution was prepared with TXA1 (purity >95%) and it was cooled at −4 °C. A solution of hydrogen chloride 2.0 M in diethyl ether solution (1 mL). An orange precipitate was formed and it was placed at −4 °C overnight. The solid thus obtained was filtered, washed with 90 mL of diethyl ether, and dried in a desiccator containing phosphorus pentoxide furnishing 1-{[2-(diethylamino)ethyl]amino}-4-propoxy-9H-thioxanthen-9-one hydrochloride (TXA1.HCl). For the in vitro experiments, a TXA1.HCl 60 mM stock solution was prepared in H_2_O and stored at −20 °C. For the xenograft experiments, a 7.5 mg/mL TXA1.HCl stock solution was prepared in saline and stored at 4 °C.

### 3.2. Cell Culture and Treatment with TXA1.HCl

The non-small lung cancer (NSCLC) cell line NCI-H460 (a kind gift from NCI, Bethesda, USA) used in this study was genotyped and routinely cultured as described in [18]. Cell number and viability were assessed with the Trypan Blue exclusion assay. For each experiment, cells were plated (1 × 10^5^ cells/well in 6-well plates), incubated for 24 h to allow adhesion, and then treated with 6.9 µM or 9.7 µM of TXA1.HCl [corresponding to the previously determined GI_50_ and GI_75_ concentrations ([18], Supplementary Figure S1)] for 24 h or 48 h, determined with the Sulforhodamine B assay as previously described in [18,20]. Control treatments consisting of untreated cells (Blank) or cells treated with equivalent volumes of H_2_O (Control) were used.

### 3.3. Mouse Xenograft Model

Animal experiments were carried out in accordance with the European Guidelines for the Care and Use of Laboratory Animals, Directive 2010/63/UE. Ethical approval was obtained from the Ethic Committee of the Faculty of Pharmacy of the University of Porto and the study was approved by Direcção Geral de Alimentação e Veterinária (DGAV), the National Entity Regulator. N:NIH(S)II-nu/nu mice, strain previously described by others [58], were produced under the supervision of IPATIMUP’s responsible staff, housed and maintained at CIM-FMUP Animal House in a pathogen-free environment under controlled conditions of light and humidity. The following Humane Endpoints for euthanasia were established: (i) any signals of distress, suffering or pain; (ii) weight loss greater than 20–25% of the body mass; (iii) anorexia and moribund state, related or not to the experimental procedure and (iv) maximum tumor volumes could not exceed 2000 mm^3^. NCI-H460 cells (5 × 10^5^ in 100 μL of non-supplemented medium) were inoculated *s.c.* into both flanks of 6-week-old male nude mice from the N:NIH(S) II-nu/nu strain. Mice were weighted and observed regularly for tumor development and signs of disease. Tumor volume was measured with a caliper and estimated using the formula W x L2 x ½, where “W” is the width and “L” is the length of the tumor. Tumor size was measured individually for mice in each group (control vs treated) at all the selected time-points. When two tumors or bi-lobulated tumors were present at the same mice (more frequent), each lobule was measured alone and the total volume inferred. Once tumors reached mean volumes of 40–80 mm^3^, treatment with TXA1.HCl (50 mg/kg) or vehicle solvent (saline) was initiated by *s.c.* injection 3 times per week. For each condition (control and TXA1.HCl treatment), seven mice were studied. At the end of the experiment (22 days after first treatment), blood was collected from mice to isolate serum for toxicology studies. Tumors were extracted, weighed and sectioned into two portions: (i) one fixed in 10% buffered formalin for subsequent paraffin embedding and histopathological and immunohistochemical analysis and (ii) the other frozen in liquid nitrogen and stored at −80 °C until further processed.

### 3.4. RNA Extraction 

Total RNA was extracted from NCI-H460 frozen xenografted cells using TRI Reagent® (Ambion, Life Technologies, Thermo Fisher Scientific, Waltham, MA USA), according to the manufacturer´s instructions. For this, tissue samples were mechanically disrupted and homogenized in TRI Reagent^®^ using a tissue homogenizer (IKA-Werke, Staufen, Germany). Total RNA (500 ng) was treated with TurboDNase I (Ambion) for genomic DNA removal. For total RNA extraction from NCI-H460 cells, the Pure Link RNA Mini kit (Thermo Fisher Scientific, Waltham, MA, USA) was used according to the manufacturer’s instructions. RNA concentration and quality were analyzed using the NanoDrop ND-1000 Spectrophotometer (Thermo Fisher Scientific, Waltham, MA, USA). For gene expression analysis, the quality of the RNA isolated from NCI-H460 cells was also verified in RNA 6000 Nano LabChip using BioAnalyzer 2100 (Agilent, Santa Clara, CA, USA).

### 3.5. Microarray cDNA Analysis 

Total RNA (100 ng) of each sample was used in GeneChip Human Genome Arrays (Affymetrix, Thermo Fisher Scientific, Waltham, MA USA), as an outsourced service to the Gene Expression Unit at “Instituto Gulbenkian de Ciência”. Data analysis included calculation of absolute values and normalization with internal standards. Microarray Data Acquisition and Analysis—RNA expression profiling was performed using the Affymetrix GeneChip^®^ technology, following the protocols recommended by the manufacturers. The mRNA expression was analyzed in the NCI-H460 cells with or without treatment with 6.9 µM TXA1.HCl for 24 h, using three biological replicas for each condition, in Affymetrix arrays (HuGENE-2_1, Probe set annotation). The data were collectively analyzed by using the R package “AFFYLMGUI” (http://www.bioconductor.org) [59]. Background adjustment was done by using robust multichip average (RMA) [60]. Correction for multiple testing was done by the method of Benjamini and Hochberg [61]. Microarray data is publicly available at Gene Expression Omnibus (GEO), with accession number GSE110825. Transcripts ≥1.5 up- or down-regulated and with an adjusted *p* value of 0.05 or less were considered to be significant. DAVID [62] and CoFRA [24] was used for functional enrichment of the significant regulated genes. 

### 3.6. Quantitative Real-time PCR

cDNA was synthesized using random hexanucleotide primers (Invitrogen, Carlsbad, CA, USA), RNase Out (Invitrogen) and M-MULV reverse transcriptase (Fermentas, Thermo Fisher Scientific, Waltham, MA, USA), as indicated by the manufacturer. Quantitative real-time PCR reactions were carried out in a 20 µL mixture with Fast SYBR Green Master Mix (Applied Biosystems, Thermo Fisher Scientific, Waltham, MA, USA) for the RNA extracted from cell lines or with KAPA SYBR Fast LowROX Master Mix (KapaBiosystems, Hoffmann-La Roche, Basel, Switzerland) for the RNA extracted from the xenografted tumors. Real-time PCR was carried out using an ABI PRISM 7500 Sequence Detection System (Applied Biosystems, Thermo Fisher Scientific, Waltham, MA USA), and reactions were run in triplicate in three independent experiments. The primer sequences and concentrations used were the following: Wipi1 (300 nM) forward 5′-TCCACGGTGCCAGGTTATTC-3′ and reverse 5′-TCTGATTTCCACGGCACAAGA-3’; HMGCS1 (150 nM) forward 5’-AAGTCCAGGCCAGCAGTGA-3′ and reverse 5′-ATATTCACAGCTCCTGAATGTACC-3’ [63], TM7SF2 (150 nM) forward 5′-CGCTTTCATCTTCAGCCTCTTT-3´ and reverse 5’-GTCGTAAATCGGATTGCCTGAG-3′; RRAGD (150 nM) forward 5’-AGGAGGGAGTTCTGGACTTCA-3’ and reverse 5′-GAATAGACGACTTGCCGCTTCT-3´; cyclin G2 (150 nM) forward 5′-CCAGAACCTCCACAACAGCTACTA-3′ and reverse 5′-TCACAAGAGTCCTCACTTTCACTTTC-3′ and Hprt1 (150 nM) forward 5′-GCAGACTTTGCTTTCCTTGGTCAG-3′and reverse 5′-GTCTGGCTTATATCCAACACTTCGTG-3’ [64]. For each set of primers, serial dilutions of NCI-H460 cells cDNA were used for the standard curve determination, and PCR efficiency was estimated as being always higher than 90%. Dissociation curves were generated. Data from the housekeeping gene Hprt1 was used as an endogenous control to normalize the expression levels of each analyzed gene [65]. 

### 3.7. Tumor Histology and Immunohistochemical Analysis

Tumors from control and treated groups previously grown in the animal model were fixed in 10% (*v*/*v*) buffered formalin for at least 48 h and routinely processed for paraffin embedding. Serial 4 μm-thick sections were cut for hematoxylin and eosin (H&E) stain and immunohistochemistry for the proliferation marker Ki-67. Briefly, the sections were incubated with anti-Ki-67 (M724001-2, 1:100, DAKO, Glostrup, Denmark) for 1 h at room temperature. Antigen retrieval was performed in citrate buffer, pH 6, according to the antibody manufacturer. Sections were then incubated with a Double stain System, Rabbit/Mouse (DAB+/Permanent Red) (DAKO, Glostrup, Denmark). Slides were examined by light microscopy in an Axioskop 2 Zeiss microscope (Carl Zeiss, Jena, Germany) and photographs acquired using a Nikon DS-L1 camera (Nikon, Tokyo, Japan) with 200× magnification. A minimum of 1000 cells were evaluated per slide. 

### 3.8. TUNEL Assay

For experiments in cultured cells, the TUNEL assay was carried out using the ‘‘in situ cell death detection kit—fluorescein’’ (Roche, Boulogne-Billancourt Cedex, France) as previously described [20,66]. For the analysis of apoptosis in tumor xenograft tissue sections, the TUNEL assay kit “Apoptag Red in situ apoptosis detection kit” (Chemicon, Temecula, CA, USA) was used as previously described [67]. Cells were observed in a DMIRE 2 fluorescence microscope (Leica, Wetzlar, Germany). A minimum of 500 or 1000 cells were counted per slide in the case of cultured cells or tissue sections, respectively. 

### 3.9. BrdU Incorporation Assay

Following 23 h treatment, the cells were further incubated with 1 µM BrdU (5′-bromodeoxyuridine, Sigma, St. Louis, Missouri, USA) for 1 h. The cells were then fixed with 4% PFA and cytospins were prepared. Following a denaturation step of 20 min with 2 M HCl, the cells were incubated with mouse anti-BrdU (1:10, Dako) and further incubated with fluorescein-labeled rabbit anti-mouse antibody (1:100, Dako), as previously described [68,69]. Slides were mounted in Vectashield Mounting Media with DAPI (Vector Laboratories Inc., Burlingame, CA, USA) and the cells were observed in a DM2000 microscope (LEICA, Wetzlar, Germany). A minimum of 500 cells were counted per slide. 

### 3.10. Filipin Staining

For the analysis of cells in culture, the cells were plated in glass coverslips for 24 h and treated with 6.9 µM or 9.7 µM TXA1.HCl for 24 or 48 h. For the analysis of xenografted tumor cells, frozen xenografted tumors were immersed in OCT compound, frozen and 10-µm-thick sections were cut. All samples were fixed in 4% paraformaldehyde in PBS, incubated with 1.5 mg/mL glycin in PBS for 10 min, washed and further incubated with 0.05 mg/mL Filipin III (Sigma, St. Louis, Missouri, USA) in PBS/10% FBS for 1 h After washing, coverslips were mounted in Vectashield Mounting Media (Vector Laboratories, Burlingame, CA, USA). The cells were observed and digital images were captured with a fluorescence microscope (ZEISS Axio Imager.Z1) coupled with the ApoTome Imaging System (Zeiss, Oberkochen, Germany). 

### 3.11. Protein Expression Analysis

Total protein lysates were prepared by lysing cells with Winman’s buffer (1% NP-40, 0.1 M Tris -HCl pH 8.0, 0.15 M NaCl and 5 mM EDTA) with protease inhibitor cocktail (Roche, Boulogne-Billancourt Cedex, France) and phosphatase inhibitor cocktail. After determination of the total protein content with the DC™ Protein Assay kit (Biorad, Hercules, CA, USA), total cell lysates (20 µg) were loaded onto a SDS-PAGE gel [67] and then transferred into nitrocellulose membranes (Amersham, GE Healthcare, Cleveland, OH, USA). Incubation with the following primary antibodies was performed: rabbit anti-Poly (ADP-ribose) polymerase (PARP-1, 1:2000, Santa Cruz Biotechnology, Heidelberg, Germany), rabbit anti-Light Chain 3 B (LC3, 1:1000, Cell Signaling, Leiden, Netherlands), mouse anti-P70 S6K (54D2; 1:1000, Cell Signaling, Leiden, Netherlands), rabbit anti-phospho P70 S6K (1:1000, Cell Signaling, Leiden, Netherlands), goat anti-Actin antibody (1:2000, Santa Cruz Biotechnology, Heidelberg, Germany) or mouse anti-Tubulin antibody (1:10000, Sigma-Aldrich, St. Louis, Missouri, EUA). The corresponding secondary antibodies were the following: goat anti-rabbit IgG-HRP, donkey anti-goat IgG-HRP or goat anti-mouse IgG-HRP (all 1:2000, Santa Cruz Biotechnology, Heidelberg, Germany). Chemiluminescence was detected using the Amersham™ ECL Western Blotting Detection Reagents (GE Healthcare, Cleveland, OH, USA), the Amersham Hyperfilm ECL (GE Healthcare, Cleveland, OH, USA) and the Kodak GBX developer and fixer (Sigma, St. Louis, Missouri, EUA), as previously described [70,71].

### 3.12. Biochemical and Toxicological Analysis

Mice serum were measured on the Roche Cobas Mira Plus automated chemistry analyzer (Roche, Massachussetts, USA) regarding the following biochemical parameters, according to standard methods: creatinine and urea, as renal function markers; creatine kinase myocardial band (CKMB) and creatine kinase (CK-R), as cardiac function markers; alanine aminotransferase (ALT) and aspartate aminotransferase (AST), as liver function markers. Data was statistically analyzed using GRAPHPAD PRISM software (La Jolla, CA, USA). Student’s *t*-test was used to test significant differences between the means of treated group vs. control group (* *p* < 0.05).

## 4. Conclusions

Overall, this study showed that TXA1.HCl upregulates steroid biosynthesis in vitro and affects the expression of proteins involved in steroid trafficking. In addition, the verification of its efficacy in vivo validated this molecule as a lead compound. Analysis of cholesterol localization both in vitro and in vivo, in xenografts from NCI-H460 cells in nude mice, showed that TXA1.HCl altered cholesterol trafficking and promoted its cytoplasmic accumulation. Altered cholesterol localization has been previously shown to result in mTOR inhibition and consequently in the induction of autophagy. As we had previously shown in other human tumor cell lines, in the present work TXA1.HCl treatment also induced autophagy in NCI-H460 cells (inhibiting the mTOR pathway as observed by the decreased phosphorylation of P70S6K). 

This study also raises new questions, particularly in what concerns the observed alterations of Ras related GTPases, namely of RagD both in vitro and in vivo. Although the exact function of Rags is still unknown, they have been described as being mainly involved in the mTOR´s lysosomal localization and activity. Additional studies will help further elucidate whether the RagC/D increase that was induced by TXA1.HCl treatment affects these or other mechanisms. 

The present work gives new insights into the mechanism of action of a lead thioxanthone compound, and supports the potential of interfering with cholesterol metabolism/trafficking as an anticancer therapeutic strategy.

## Figures and Tables

**Table 1:** Blood biochemical analyses of treated and control mice.

	Creatinine (mg/dL)	Urea (mg/dL)	Total Protein (g/dL)	CKMB (U/L)	AST (U/L)	ALT (U/L)
**Control**	0.17 ± 0.03	48.63 ± 2.17	4.86 ± 0.30	128.00 ± 15.27	221.50 ± 30.85	29.67 ± 0.88
**TXA1.HCl (50 mg/kg)**	0.27 ± 0.10	49.58 ± 5.28	5.65 ± 0.69	151.50 ± 28.60	230.20 ± 57.9	38.25 ± 6.05

Evaluations in the serum of NCI-H460 xenografted mice after treatment with saline (as control) or with TXA1.HCl at a dose of 50 mg/kg body weight. Results are expressed as mean ± SEM (*n* = 3 to 6). No statistically significant differences (* *p* < 0.05) were observed between groups. CKMB, creatine kinase myocardial band; ALT, alanine aminotransferase; AST, aspartate aminotransferase.

## Supplementary Materials

The following are available online at http://www.mdpi.com/1420-3049/23/12/3301/s1. Table S1. Gene expression alterations in NCI-H460 cells induced by TXA1.HCl treatment; Figure S1. Dose-response curve for TXA1.HCl in NCI-H460 cells, analyzed with the sulphorodamine B assay; Figure S2. Tumor weight after treatment with TXA1.HCl.
