# Supplementary material for: The Antitumor Activity of a Lead Thioxanthone is Associated with Alterations in Cholesterol Localization
Source: Molecules. 2018 Dec 12;23(12):3301. doi: 10.3390/molecules23123301 (PMC6321308; doi:10.3390/molecules23123301)
Supplement: Supplementary file 1 [file molecules-23-03301-s001.pdf]

Supplementary Material

The Antitumor Activity of a Lead Thioxanthone is Associated with Alterations in Cholesterol Localization.

Raquel T. Lima, Diana Sousa, Ana Sara Gomes, Nuno Mendes, Rune Matthiesen, Madalena Pedro, Franklim Marques, Madalena M. Pinto, Emília Sousa, M. Helena Vasconcelos

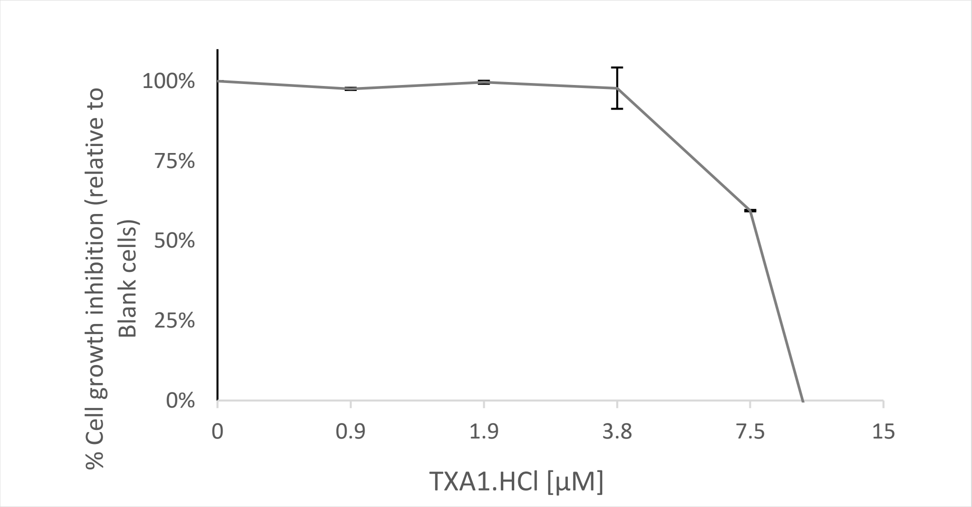

Figure S1. Dose-response curve for TXA1.HCl in NCI-H460 cells, analyzed with the sulphorodamine B assay. Cells were treated for 48 h with increasing concentrations of TXA1.HCl. Cell growth inhibition is expressed as % of Blank cells (cells treated with medium only). Results are expressed as the mean ± SEM of at least three independent experiments. The maximum vehicle (saline) concentration was also analysed, as a control, and had no effect on cell growth (*data not shown).*

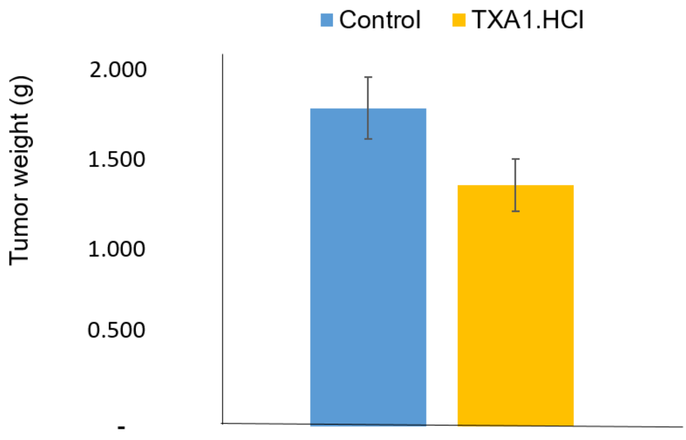

Figure S2. Tumor weight after treatment with TXA1.HCl**.** Nude mice bearing tumor xenografts (40-80 mm3) were injected 3 times per week with TXA1.HCl (50 mg/kg, *n* = 7) or vehicle control (saline, *n* = 7). After 22 days, tumors were resected and their weight was measured. Results are expressed as the mean ± SEM (*n* = 7).

Table S1 Gene expression alterations in NCI-H460 cells induced by TXA1.HCl treatment.

	Symbol	Description	p.adjusted	Fold Change	
**3157_at**	**HMGCS1**	**3-hydroxy-3-methylglutaryl-CoA synthase 1 (soluble) [Source:HGNC Symbol;Acc:5007]**	0.000065	**+**	**4.45**
58528_at	RRAGD	Ras-related GTP binding D [Source:HGNC Symbol;Acc:19903]	0.000364	+	3.82
9388_at	LIPG	lipase, endothelial [Source:HGNC Symbol;Acc:6623]	0.000364	+	3.58
3638_at	INSIG1	insulin induced gene 1 [Source:HGNC Symbol;Acc:6083]	0.000137	+	3.26
9201_at	DCLK1	doublecortin-like kinase 1 [Source:HGNC Symbol;Acc:2700]	0.000095	+	3.11
9518_at	GDF15	growth differentiation factor 15 [Source:HGNC Symbol;Acc:30142]	0.000065	+	2.91
4312_at	MMP1	matrix metallopeptidase 1 (interstitial collagenase) [Source:HGNC Symbol;Acc:7155]	0.00007	+	2.89
4758_at	NEU1	sialidase 1 (lysosomal sialidase) [Source:HGNC Symbol;Acc:7758]	0.000137	+	2.83
**3156_at**	**HMGCR**	**3-hydroxy-3-methylglutaryl-CoA reductase [Source:HGNC Symbol;Acc:5006]**	0.000065	**+**	**2.74**
80339_at	PNPLA3	patatin-like phospholipase domain containing 3 [Source:HGNC Symbol;Acc:18590]	0.001397	+	2.69
230_at	ALDOC	aldolase C, fructose-bisphosphate [Source:HGNC Symbol;Acc:418]	0.000168	+	2.60
**4597_at**	**MVD**	**mevalonate (diphospho) decarboxylase [Source:HGNC Symbol;Acc:7529]**	0.000148	**+**	**2.57**
255394_at	TCP11L2	t-complex 11, testis-specific-like 2 [Source:HGNC Symbol;Acc:28627]	0.001397	+	2.54
55902_at	ACSS2	acyl-CoA synthetase short-chain family member 2 [Source:HGNC Symbol;Acc:15814]	0.000974	+	2.47
54800_at	KLHL24	kelch-like family member 24 [Source:HGNC Symbol;Acc:25947]	0.001407	+	2.46
**6307_at**	**MSMO1**	**methylsterol monooxygenase 1 [Source:HGNC Symbol;Acc:10545]**	0.00009	**+**	**2.46**
**7108_at**	**TM7SF2**	**transmembrane 7 superfamily member 2 [Source:HGNC Symbol;Acc:11863]**	0.004175	**+**	**2.43**
5208_at	PFKFB2	6-phosphofructo-2-kinase/fructose-2,6-biphosphatase 2 [Source:HGNC Symbol;Acc:8873]	0.00109	+	2.41
**6713_at**	**SQLE**	**squalene epoxidase [Source:HGNC Symbol;Acc:11279]**	0.000137	**+**	**2.32**
11082_at	ESM1	endothelial cell-specific molecule 1 [Source:HGNC Symbol;Acc:3466]	0.003379	+	2.31
**4047_at**	**LSS**	**lanosterol synthase (2,3-oxidosqualene-lanosterol cyclase) [Source:HGNC Symbol;Acc:6708]**	0.000168	**+**	**2.30**
1958_at	EGR1	early growth response 1 [Source:HGNC Symbol;Acc:3238]	0.000148	+	2.28
4856_at	NOV	nephroblastoma overexpressed [Source:HGNC Symbol;Acc:7885]	0.000754	+	2.24
3373_at	HYAL1	hyaluronoglucosaminidase 1 [Source:HGNC Symbol;Acc:5320]	0.001115	+	2.21
2919_at	CXCL1	chemokine (C-X-C motif) ligand 1 (melanoma growth stimulating activity, alpha) [Source:HGNC Symbol;Acc:4602]	0.004308	+	2.18
**4598_at**	**MVK**	**mevalonate kinase [Source:HGNC Symbol;Acc:7530]**	0.000807	**+**	**2.14**
55062_at	WIPI1	WD repeat domain, phosphoinositide interacting 1 [Source:HGNC Symbol;Acc:25471]	0.00109	+	2.13

9245_at	GCNT3	glucosaminyl (N-acetyl) transferase 3, mucin type [Source:HGNC Symbol;Acc:4205]	0.003756	+	2.12
**1718_at**	**DHCR24**	**24-dehydrocholesterol reductase [Source:HGNC Symbol;Acc:2859]**	0.000148	**+**	**2.11**
**3422_at**	**IDI1**	**isopentenyl-diphosphate delta isomerase 1 [Source:HGNC Symbol;Acc:5387]**	0.000653	**+**	**2.10**
**39_at**	**ACAT2**	**acetyl-CoA acetyltransferase 2 [Source:HGNC Symbol;Acc:94]**	0.000148	**+**	**2.10**
80274_at	SCUBE1	signal peptide, CUB domain, EGF-like 1 [Source:HGNC Symbol;Acc:13441]	0.000549	+	2.08
9415_at	FADS2	fatty acid desaturase 2 [Source:HGNC Symbol;Acc:3575]	0.002159	+	2.06
2180_at	ACSL1	acyl-CoA synthetase long-chain family member 1 [Source:HGNC Symbol;Acc:3569]	0.000818	+	2.06
728228_at	RP4-779E11.3		0.011305	+	2.05
112464_at	PRKCDBP	protein kinase C, delta binding protein [Source:HGNC Symbol;Acc:9400]	0.002591	+	2.03
7357_at	UGCG	UDP-glucose ceramide glucosyltransferase [Source:HGNC Symbol;Acc:12524]	0.001136	+	2.01
3725_at	JUN	jun proto-oncogene [Source:HGNC Symbol;Acc:6204]	0.001397	+	2.01
197187_at	SNAI3-AS1	SNAI3 antisense RNA 1 [Source:HGNC Symbol;Acc:28327]	0.011874	+	2.00
201799_at	TMEM154	transmembrane protein 154 [Source:HGNC Symbol;Acc:26489]	0.015656	+	1.99
**1595_at**	**CYP51A1**	**cytochrome P450, family 51, subfamily A, polypeptide 1 [Source:HGNC Symbol;Acc:2649]**	0.001585	**+**	**1.97**
79152_at	FA2H	fatty acid 2-hydroxylase [Source:HGNC Symbol;Acc:21197]	0.001275	+	1.93
201232_at	SLC16A13	solute carrier family 16, member 13 (monocarboxylic acid transporter 13) [Source:HGNC Symbol;Acc:31037]	0.002473	+	1.93
**2224_at**	**FDPS**	**farnesyl diphosphate synthase [Source:HGNC Symbol;Acc:3631]**	0.005014	**+**	**1.91**
64109_at	CRLF2	cytokine receptor-like factor 2 [Source:HGNC Symbol;Acc:14281]	0.004709	+	1.91
4923_at	NTSR1	neurotensin receptor 1 (high affinity) [Source:HGNC Symbol;Acc:8039]	0.003909	+	1.90
93129_at	ORAI3	ORAI calcium release-activated calcium modulator 3 [Source:HGNC Symbol;Acc:28185]	0.005522	+	1.90
11022_at	TDRKH	tudor and KH domain containing [Source:HGNC Symbol;Acc:11713]	0.001136	+	1.88
6038_at	RNASE4	ribonuclease, RNase A family, 4 [Source:HGNC Symbol;Acc:10047]	0.001468	+	1.88
10577_at	NPC2	Niemann-Pick disease, type C2 [Source:HGNC Symbol;Acc:14537]	0.001893	+	1.87
1649_at	DDIT3	DNA-damage-inducible transcript 3 [Source:HGNC Symbol;Acc:2726]	0.001468	+	1.86
253039_at	PSMD5-AS1	PSMD5 antisense RNA 1 (head to head) [Source:HGNC Symbol;Acc:27367]	0.010868	+	1.84
2026_at	ENO2	enolase 2 (gamma, neuronal) [Source:HGNC Symbol;Acc:3353]	0.001468	+	1.83
3949_at	LDLR	low density lipoprotein receptor [Source:HGNC Symbol;Acc:6547]	0.000464	+	1.82
5743_at	PTGS2	prostaglandin-endoperoxide synthase 2 (prostaglandin G/H synthase and cyclooxygenase) [Source:HGNC Symbol;Acc:9605]	0.016853	+	1.82
79888_at	LPCAT1	lysophosphatidylcholine acyltransferase 1 [Source:HGNC Symbol;Acc:25718]	0.000818	+	1.80

**2222_at**	**FDFT1**	**farnesyl-diphosphate farnesyltransferase 1 [Source:HGNC Symbol;Acc:3629]**	0.001397	**+**	**1.80**
10493_at	VAT1	vesicle amine transport protein 1 homolog (T. californica) [Source:HGNC Symbol;Acc:16919]	0.000754	+	1.79
79071_at	ELOVL6	ELOVL fatty acid elongase 6 [Source:HGNC Symbol;Acc:15829]	0.000951	+	1.79
11221_at	DUSP10	dual specificity phosphatase 10 [Source:HGNC Symbol;Acc:3065]	0.002159	+	1.78
170393_at	C10orf91	chromosome 10 open reading frame 91 [Source:HGNC Symbol;Acc:27275]	0.019345	+	1.77
23475_at	QPRT	quinolinate phosphoribosyltransferase [Source:HGNC Symbol;Acc:9755]	0.007715	+	1.77
4084_at	MXD1	MAX dimerization protein 1 [Source:HGNC Symbol;Acc:6761]	0.001397	+	1.75
2896_at	GRN	granulin [Source:HGNC Symbol;Acc:4601]	0.001136	+	1.75
978_at	CDA	cytidine deaminase [Source:HGNC Symbol;Acc:1712]	0.011368	+	1.75
64342_at	HS1BP3	HCLS1 binding protein 3 [Source:HGNC Symbol;Acc:24979]	0.001577	+	1.75
326625_at	MMAB	methylmalonic aciduria (cobalamin deficiency) cblB type [Source:HGNC Symbol;Acc:19331]	0.002473	+	1.74
6809_at	STX3	syntaxin 3 [Source:HGNC Symbol;Acc:11438]	0.007715	+	1.74
**6309_at**	**SC5D**	**sterol-C5-desaturase [Source:HGNC Symbol;Acc:10547]**	0.001577	**+**	**1.73**
2706_at	GJB2	gap junction protein, beta 2, 26kDa [Source:HGNC Symbol;Acc:4284]	0.005976	+	1.73
23558_at	WBP2	WW domain binding protein 2 [Source:HGNC Symbol;Acc:12738]	0.006812	+	1.73
1543_at	CYP1A1	cytochrome P450, family 1, subfamily A, polypeptide 1 [Source:HGNC Symbol;Acc:2595]	0.001397	+	1.72
84002_at	B3GNT5	UDP-GlcNAc:betaGal beta-1,3-N-acetylglucosaminyltransferase 5 [Source:HGNC Symbol;Acc:15684]	0.004175	+	1.71
100129518_at	SOD2	superoxide dismutase 2, mitochondrial [Source:HGNC Symbol;Acc:11180]	0.001436	+	1.71
145788_at	C15orf65	chromosome 15 open reading frame 65 [Source:HGNC Symbol;Acc:44654]	0.008228	+	1.71
**1717_at**	**DHCR7**	**7-dehydrocholesterol reductase [Source:HGNC Symbol;Acc:2860]**	0.001945	**+**	**1.70**
79772_at	MCTP1	multiple C2 domains, transmembrane 1 [Source:HGNC Symbol;Acc:26183]	0.003909	+	1.69
427_at	ASAH1	N-acylsphingosine amidohydrolase (acid ceramidase) 1 [Source:HGNC Symbol;Acc:735]	0.000818	+	1.69
96459_at	FNIP1	folliculin interacting protein 1 [Source:HGNC Symbol;Acc:29418]	0.001407	+	1.68
901_at	CCNG2	cyclin G2 [Source:HGNC Symbol;Acc:1593]	0.001961	+	1.68
3576_at	IL8	interleukin 8 [Source:HGNC Symbol;Acc:6025]	0.005943	+	1.68
8303_at	SNN	stannin [Source:HGNC Symbol;Acc:11149]	0.006699	+	1.67
10263_at	CDK2AP2	cyclin-dependent kinase 2 associated protein 2 [Source:HGNC Symbol;Acc:30833]	0.010571	+	1.67
6275_at	S100A4	S100 calcium binding protein A4 [Source:HGNC Symbol;Acc:10494]	0.013825	+	1.66
374383_at	NCR3LG1	natural killer cell cytotoxicity receptor 3 ligand 1 [Source:HGNC Symbol;Acc:42400]	0.009915	+	1.66
1200_at	TPP1	tripeptidyl peptidase I [Source:HGNC Symbol;Acc:2073]	0.004175	+	1.66

51171_at	HSD17B14	hydroxysteroid (17-beta) dehydrogenase 14 [Source:HGNC Symbol;Acc:23238]	0.02697	+	1.65
90271_at	NA	NA	0.018339	+	1.65
26503_at	SLC17A5	solute carrier family 17 (anion/sugar transporter), member 5 [Source:HGNC Symbol;Acc:10933]	0.002429	+	1.65
3552_at	IL1A	interleukin 1, alpha [Source:HGNC Symbol;Acc:5991]	0.005976	+	1.64
7111_at	TMOD1	tropomodulin 1 [Source:HGNC Symbol;Acc:11871]	0.002591	+	1.64
11182_at	SLC2A6	solute carrier family 2 (facilitated glucose transporter), member 6 [Source:HGNC Symbol;Acc:11011]	0.028888	+	1.64
7128_at	TNFAIP3	tumor necrosis factor, alpha-induced protein 3 [Source:HGNC Symbol;Acc:11896]	0.002599	+	1.63
79639_at	TMEM53	transmembrane protein 53 [Source:HGNC Symbol;Acc:26186]	0.014775	+	1.62
23175_at	LPIN1	lipin 1 [Source:HGNC Symbol;Acc:13345]	0.001241	+	1.62
1185_at	CLCN6	chloride channel, voltage-sensitive 6 [Source:HGNC Symbol;Acc:2024]	0.002917	+	1.62
5833_at	PCYT2	phosphate cytidylyltransferase 2, ethanolamine [Source:HGNC Symbol;Acc:8756]	0.001774	+	1.62
4881_at	NPR1	natriuretic peptide receptor A/guanylate cyclase A (atrionatriuretic peptide receptor A) [Source:HGNC Symbol;Acc:7943]	0.037329	+	1.61
5909_at	RAP1GAP	RAP1 GTPase activating protein [Source:HGNC Symbol;Acc:9858]	0.007366	+	1.61
55747_at	FAM21B	family with sequence similarity 21, member B [Source:HGNC Symbol;Acc:23417]	0.016853	+	1.61
2760_at	GM2A	GM2 ganglioside activator [Source:HGNC Symbol;Acc:4367]	0.027387	+	1.61
153830_at	RNF145	ring finger protein 145 [Source:HGNC Symbol;Acc:20853]	0.001275	+	1.60
158160_at	HSD17B7P2	hydroxysteroid (17-beta) dehydrogenase 7 pseudogene 2 [Source:HGNC Symbol;Acc:28120]	0.037017	+	1.59
399694_at	SHC4	SHC (Src homology 2 domain containing) family, member 4 [Source:HGNC Symbol;Acc:16743]	0.024048	+	1.59
64121_at	RRAGC	Ras-related GTP binding C [Source:HGNC Symbol;Acc:19902]	0.002872	+	1.59
3382_at	ICA1	islet cell autoantigen 1, 69kDa [Source:HGNC Symbol;Acc:5343]	0.006699	+	1.58
113146_at	AHNAK2	AHNAK nucleoprotein 2 [Source:HGNC Symbol;Acc:20125]	0.003501	+	1.58
10468_at	FST	follistatin [Source:HGNC Symbol;Acc:3971]	0.008094	+	1.57
90809_at	TMEM55B	transmembrane protein 55B [Source:HGNC Symbol;Acc:19299]	0.002159	+	1.57
3992_at	FADS1	fatty acid desaturase 1 [Source:HGNC Symbol;Acc:3574]	0.001397	+	1.56
6550_at	SLC9A3	solute carrier family 9, subfamily A (NHE3, cation proton antiporter 3), member 3 [Source:HGNC Symbol;Acc:11073]	0.022653	+	1.56
9586_at	CREB5	cAMP responsive element binding protein 5 [Source:HGNC Symbol;Acc:16844]	0.039378	+	1.55
50640_at	PNPLA8	patatin-like phospholipase domain containing 8 [Source:HGNC Symbol;Acc:28900]	0.006044	+	1.55
222487_at	GPR97	G protein-coupled receptor 97 [Source:HGNC Symbol;Acc:13728]	0.013261	+	1.55
3164_at	NR4A1	nuclear receptor subfamily 4, group A, member 1 [Source:HGNC Symbol;Acc:7980]	0.037426	+	1.55
**50814_at**	**NSDHL**	**NAD(P) dependent steroid dehydrogenase-like [Source:HGNC Symbol;Acc:13398]**	0.004175	**+**	**1.55**

9122_at	SLC16A4	solute carrier family 16, member 4 (monocarboxylic acid transporter 5) [Source:HGNC Symbol;Acc:10925]	0.002795	+	1.55
8320_at	EOMES	eomesodermin [Source:HGNC Symbol;Acc:3372]	0.009223	+	1.54
3727_at	JUND	jun D proto-oncogene [Source:HGNC Symbol;Acc:6206]	0.002429	+	1.54
140576_at	S100A16	S100 calcium binding protein A16 [Source:HGNC Symbol;Acc:20441]	0.005356	+	1.54
22933_at	SIRT2	sirtuin 2 [Source:HGNC Symbol;Acc:10886]	0.006853	+	1.54
6721_at	SREBF2	sterol regulatory element binding transcription factor 2 [Source:HGNC Symbol;Acc:11290]	0.003379	+	1.53
56204_at	FAM214A	family with sequence similarity 214, member A [Source:HGNC Symbol;Acc:25609]	0.014109	+	1.53
286343_at	LURAP1L	leucine rich adaptor protein 1-like [Source:HGNC Symbol;Acc:31452]	0.006075	+	1.52
2034_at	EPAS1	endothelial PAS domain protein 1 [Source:HGNC Symbol;Acc:3374]	0.002716	+	1.52
84418_at	CYSTM1	cysteine-rich transmembrane module containing 1 [Source:HGNC Symbol;Acc:30239]	0.007715	+	1.52
51646_at	YPEL5	yippee-like 5 (Drosophila) [Source:HGNC Symbol;Acc:18329]	0.027319	+	1.51
729096_at	RP11-574K11.28		0.046427	+	1.51
353189_at	SLCO4C1	solute carrier organic anion transporter family, member 4C1 [Source:HGNC Symbol;Acc:23612]	0.047958	+	1.51
112483_at	SAT2	spermidine/spermine N1-acetyltransferase family member 2 [Source:HGNC Symbol;Acc:23160]	0.012976	+	1.51
22795_at	NID2	nidogen 2 (osteonidogen) [Source:HGNC Symbol;Acc:13389]	0.002591	+	1.51
134429_at	STARD4	StAR-related lipid transfer (START) domain containing 4 [Source:HGNC Symbol;Acc:18058]	0.002574	+	1.50
100128077_at	NA	NA	0.010873	+	1.50
94241_at	TP53INP1	tumor protein p53 inducible nuclear protein 1 [Source:HGNC Symbol;Acc:18022]	0.045657	+	1.50
84962_at	AJUBA	ajuba LIM protein [Source:HGNC Symbol;Acc:20250]	0.025389	-	1.51
100616214_at	MIR4667	microRNA 4667 [Source:HGNC Symbol;Acc:41723]	0.01897	-	1.53
64759_at	TNS3	tensin 3 [Source:HGNC Symbol;Acc:21616]	0.001936	-	1.53
6775_at	STAT4	signal transducer and activator of transcription 4 [Source:HGNC Symbol;Acc:11365]	0.02307	-	1.54
57211_at	GPR126	G protein-coupled receptor 126 [Source:HGNC Symbol;Acc:13841]	0.004308	-	1.56
26251_at	KCNG2	potassium voltage-gated channel, subfamily G, member 2 [Source:HGNC Symbol;Acc:6249]	0.021629	-	1.56
306_at	ANXA3	annexin A3 [Source:HGNC Symbol;Acc:541]	0.003137	-	1.57
56670_at	SUCNR1	succinate receptor 1 [Source:HGNC Symbol;Acc:4542]	0.001615	-	1.59
22943_at	DKK1	dickkopf WNT signaling pathway inhibitor 1 [Source:HGNC Symbol;Acc:2891]	0.011334	-	1.61
8908_at	GYG2	glycogenin 2 [Source:HGNC Symbol;Acc:4700]	0.012667	-	1.61
10769_at	PLK2	polo-like kinase 2 [Source:HGNC Symbol;Acc:19699]	0.013326	-	1.61
30832_at	ZNF354C	zinc finger protein 354C [Source:HGNC Symbol;Acc:16736]	0.021783	-	1.62

51421_at	AMOTL2	angiomotin like 2 [Source:HGNC Symbol;Acc:17812]	0.004566	-	1.65
7091_at	TLE4	transducin-like enhancer of split 4 (E(sp1) homolog, Drosophila) [Source:HGNC Symbol;Acc:11840]	0.023041	-	1.66
7052_at	TGM2	transglutaminase 2 [Source:HGNC Symbol;Acc:11778]	0.001265	-	1.67
27063_at	ANKRD1	ankyrin repeat domain 1 (cardiac muscle) [Source:HGNC Symbol;Acc:15819]	0.008632	-	1.68
26287_at	ANKRD2	ankyrin repeat domain 2 (stretch responsive muscle) [Source:HGNC Symbol;Acc:495]	0.002591	-	1.79
29015_at	SLC43A3	solute carrier family 43, member 3 [Source:HGNC Symbol;Acc:17466]	0.02697	-	1.79
3486_at	IGFBP3	insulin-like growth factor binding protein 3 [Source:HGNC Symbol;Acc:5472]	0.003243	-	1.79
1906_at	EDN1	endothelin 1 [Source:HGNC Symbol;Acc:3176]	0.004655	-	1.79
100287477_at	NA	NA	0.009821	-	1.80
3491_at	CYR61	cysteine-rich, angiogenic inducer, 61 [Source:HGNC Symbol;Acc:2654]	0.00109	-	1.82
9619_at	ABCG1	ATP-binding cassette, sub-family G (WHITE), member 1 [Source:HGNC Symbol;Acc:73]	0.011874	-	1.85
27092_at	CACNG4	calcium channel, voltage-dependent, gamma subunit 4 [Source:HGNC Symbol;Acc:1408]	0.002468	-	1.88
57393_at	TMEM27	transmembrane protein 27 [Source:HGNC Symbol;Acc:29437]	0.0005	-	1.92
794_at	CALB2	calbindin 2 [Source:HGNC Symbol;Acc:1435]	0.012946	-	1.97
7057_at	THBS1	thrombospondin 1 [Source:HGNC Symbol;Acc:11785]	0.001397	-	2.29
56256_at	SERTAD4	SERTA domain containing 4 [Source:HGNC Symbol;Acc:25236]	0.006075	-	2.42

NCI-H460 cells were treated for 24 h with 6.9 μM TXA1.HCl or water (control) and differentially expressed genes were identified. Values represent the log2 and fold change observed in gene expression in TXA1.HCl cells when compared with the control. Only genes which are upregulated or downregulated in TXA1.HCl treated cells when compared to the control (≥1.5-fold and p value of ≤ 0.05) are represented. Among these genes, the ones involved in the terpenoid and steroid biosynthesis pathways are written in bold letters.
